# The Role of the Robot in the Chemical Laboratory

**DOI:** 10.6028/jres.093.039

**Published:** 1988-06-01

**Authors:** C. H. Lochmüller

**Affiliations:** The Department of Chemistry, Duke University, Durham, NC 27706

The robot, a natural extension of the development of automation in the chemical laboratory, has recently enjoyed a flowering of interest. Robotics provides the missing link for the complete automation of standardized and new procedures by allowing the instrumentalist the option of mass-moving within the envelope of a laboratory experiment. It must be made clear that robotics is not a separate area but simply a sub-discipline within the general area of chemical instrumentation. Nothing that is done by a robot could not be done by a piece of “hard” instrumentation. The advantage of the robot is that it is “soft” instrumentation [1]. Soft instrumentation permits the laboratory worker to reprogram the manipulations carried out by automated equipment when a change is necessary in an established protocol.

Robotics has enjoyed a burst of interest because there are many established analytical procedures which involve substantial human interaction and which depend for their precision on the adaptation of humans to a protocol for a given task. It is the common experience of many laboratory managers that a technician requires a significant period of time to develop the technique required for good precision. This period of attainment of good precision is followed by a period of acceptable performance. If the task is a monotonous one, then the period of good performance is often followed by a decline in precision resulting from a growing human disinterest in the task itself. Robots provide a disinterested approach to the performance of such routine tasks and their training period differs little from the training period of a human operator.

Many administrators and laboratory managers are disappointed that robots require a significant period of time to be integrated into the laboratory environment. Robots, operating as they do as one-, or, at best, two-armed mechanical laboratory assistants incapable of vision and with limited sense of touch, require the conversion of human-assisted laboratory procedures to robot-assisted laboratory procedures and this requires training. This training often involves personnel of equivalent or higher level than would normally be assigned to the task that will be ultimately robotized. In fact, it is not unusual that the first robot installation of a kind will require 3 to 4 months of continuous effort by a team of two or three laboratory personnel, usually including one Ph.D., to be successfully implemented.

The whole problem is not the easy implementation of the number of steps required in the human- assisted example but rather the validation at every step carried out by this essentially blind, one armed, limited sensual capability laboratory assistant, the robot. In fact, most laboratory procedures, even those involving 10 or more steps, can be implemented in a laboratory using a robot within 10 days. The additional time of 4 months involves establishing those validation steps so critical to insuring that what this mindless assistant does can be traced and that anomalous results can be explained. It is this additional work that generates a sense of frustration in some laboratory personnel attempting to introduce robotics.

The more human a current robotic system is the less flexible it is. Human laboratory systems are currently defined in marketing terms as those which are essentially “turn key” systems which require little if any training by the laboratory personnel in order to perform routine tasks. Examples of this include the “PYE” approach of Zymark and the more recent contributions of Waters. Many routine sample separation operations can indeed be programmed with relative ease in either of these environments. However, robotics can contribute much more to laboratory exercise than the routine unit operations of filtration, extraction, etc. [2]. It is possible to train the robot to operate human-engineered machinery including a keyboard so that during the off hours when the robot is working alone in the laboratory it can, by access to expert systems, modify analytical procedures within established limits in order to restore analytical capability and, therefore, maintain productivity [2].

This paper, while reviewing progress in the introduction of robotics in the laboratory, will also illustrate the inclusion of various elements that are beyond the routine sample preparation operation in nature and which includes optimization of analytical conditions and referral to residing experts systems for decisions related to the next best test to perform [3]. It is true that 99% of current robot installations perform routine tasks which could be easily described by decision trees or flow programming. It is also true that robotic installations of the future, or broadly defined as simply the mass-moving component of current robotic systems, will make decisions based on intelligence bases which will involve an almost cybernetic or “clever” decision basis. Attempts to extrapolate current capability into future capability will be made.

## References

[1] Lochmüller CH, Lung KR (1986). Anal Chim Acta.

[2] Lochmüller CH, Lung KR (1986). J Liq Chrom.

[3] Lochmüller CH, Lloyd TL, Lung KR, Kahlurand M (1987). Anal Lett.

